# A lack of reproductive agency in facility-based births makes home births a first choice regardless of potential risks and medical needs—a qualitative study among multiparous women in Somaliland

**DOI:** 10.1080/16549716.2022.2054110

**Published:** 2022-04-07

**Authors:** Jama Ali Egal, Amina Essa, Rahma Yusuf, Fatumo Osman, Derie Ereg, Marie Klingberg-Allvin, Kerstin Erlandsson

**Affiliations:** a Lead Department of Midwifery, College of Medicine & Health Sciences, University of Hargeisa, Hargeisa, Somaliland; b Institution for Health and Welfare, Dalarna University, Falun, Sweden; c College of Medicine and Health Sciences, University of Hargeisa, Hargeisa, Somaliland

**Keywords:** Home-based birth, facility-based birth, decision-making, reproductive agency

## Abstract

**Background:**

Around 20% of births in Somaliland take place at health facilities staffed by trained healthcare professionals; 80% take place at home assisted by Traditional Birth Attendants (TBAs) with no formal training. There has been no research into women’s choice of place of birth.

**Objective:**

In this study, we explore multipara women’s needs and preferences when choosing the place of birth.

**Method:**

An explorative qualitative study using individual in-depth interviews analysed inductively using content analysis. The interviews were conducted in Somaliland with 25 multiparous women who had experience of giving birth both at home and at a health facility within the past three years.

**Results:**

The results provide a description of how, for women in Somaliland, a lack of reproductive agency in facility-based births makes home births a first choice regardless of potential risks and medical need. The women in this study desired intentionality in their role as mothers and sought some measure of control over the environment where they planned to give birth, depending on the circumstances of that particular birth. The results describe what quality care means for multipara women in Somaliland and how women choose birthplace based on previous experiences of care. The expectation of respectful care was a vital part for women when choosing a place of birth.

**Conclusion:**

To meet women’s needs and preferences in Somaliland, further investments are needed to strengthen the midwifery profession and to define and test a context specific midwife-led continuity of care model to be scaled up. A dialogue to create new roles and responsibilities for the TBAs who attend most home births is further needed to link them to the formal healthcare system and assure timely healthcare seeking during pregnancy and birth.

## Background

It is well established that it can be difficult for women in fragile contexts to access adequate maternal healthcare. The idea that maternal mortality could be lowered if women had better access to maternal healthcare services is one that has not been properly examined [1,2]. The WHO has estimated that 830 women die every day globally because of preventable obstetric complications. Ninety-nine per cent of these deaths occur in low- and middle-income countries of which more than half take place in sub-Saharan Africa [3]. To achieve a reduction in maternal mortality, women need to be able to access health facilities run by trained healthcare professionals that deliver evidence-based practice and uphold quality and respect in every aspect of the care provision [4].

A healthcare professional in this article is defined as an accredited and skilled birth attendant with the potential to make a positive intervention if an emergency takes place in the birth process. These Skilled Birth Attendants (SBAs), the collective term for midwives, nurses or physicians who have received formal education and training, have a tested proficiency in the skills needed to manage both normal and complicated pregnancies, births and follow-ups through the identification, management and timely referral of women and newborns for specialist treatment [5–7].

Home-based birth is common around the world [8–12]. In low- and middle-income countries, the home birth rate is often more than 50% whilst in high-income countries, the home birth rate is no more than 1–3%. In low- and middle-income countries, home births are often assisted by Traditional Birth Attendants (TBAs), local women who have developed an expertise in assisting with births but have no formal medical training. While there is growing interest in the role of the non-medical doula in some Western circles, in most high-income countries, women who choose a home birth are supported by trained midwives [13–15].

*The Lancet*’s series of articles on midwifery care (2014) focused on childbearing women’s rights and needs and the evidence-based intervention needed to secure safe births. The conclusions were presented in a framework for quality maternal and newborn care (QMNC) [16]. Midwives trained according to international standards and working in interdisciplinary teams have been identified as central to women’s maternal healthcare needs [16] and as having the potential to avert 83% of all maternal deaths globally [17]. Further evidence shows that women who receive models of midwife-led continuity of care are less likely to experience intervention and more likely to be satisfied with their care compared with women who receive other models of care [18]. Around 20% of the births in Somaliland take place at health facilities staffed by skilled healthcare professionals; 80% take place at home, assisted by TBAs with no formal training. In Somaliland, midwives based in health facilities do not carry out antenatal or postnatal visits. Thus, there is no link between the community and the maternity services [19]. At present, home births are attended by untrained TBAs. Their involvement can delay the referral of complicated cases and thus, by extension, can contribute to high maternal morbidity. TBAs act in contravention of the recommendations of the Somaliland health authorities and UN agencies [19,20]. The Somaliland government has invested in educating midwives [19,21]. Despite these efforts the mortality rate is still high, with 396 maternal deaths per 100,000 live births [3,21]. This figure is largely attributable to the shortage of midwives, equipment, and transport infrastructure within the maternity services when complications occur [15,21]. Innovative approaches are required to save the lives of women and newborns, educate healthcare professionals, particularly midwives, and strengthen their capacity [22,23]. The first step in addressing this challenge is to understand women’s choice of place of birth in a subsequent pregnancy. There is no previous research on women’s choices in relation to place of birth in Somaliland.

In this study, we explore the needs and preferences of multipara women in Somaliland when choosing place of birth.

## Methodology

### Design

This study is an exploratory qualitative study using individual in-depth interviews of multipara women (n = 25) that were then analysed inductively using qualitative content analysis according to Elo et al. [24]. Ethical approval was obtained from the Somaliland MOHD and the research ethics committee at the University of Hargeisa. Dr: CS/41105/18.

### Participants

In the *preparation phase* [24], eligible multipara women from Ahmed Dhagah community with experience of giving birth were identified as meeting the inclusion criteria for participation in this study. The criteria for inclusion was a multiparous woman who had experienced giving birth at home and at a health facility within the last three years. Twenty-six women voluntarily agreed to take part in the study. One woman with experience of home birth was excluded when she refused to be audio recorded. The interviews were conducted with 25 women between January 2015 and September 2019. For the socio-demographic details of the participants, see Table 1.

**Table 1.** Participants’ sociodemographic information

Female participants	N = 25
Age	22–38 (M = 31)
Marital status	
Married	24
Separated	1
Educational level	
Illiterate	12
Primary school	2
Intermediate	4
Lower secondary	3
Quran school	4
Occupation	
Housewife	23
Employed	2

At the time, the participants lived in the Ahmed Dhagah district of Hargeisa, the capital city of Somaliland. The district contains eight small villages with an estimated population of 23,000 inhabitants. Healthcare is managed from one large hospital and four health facilities where, at the latter, the care is free of charge. Many of the women in this district were originally Internally Displaced People who had moved to the area as a result of the civil war in the 1990s. Many of them have a low socio-economic status and are considered vulnerable [19].

### Data collection

Using a purposeful sampling approach [25], women who had experienced both types of birth setting and were able to reflect on their previous experiences, were approached by midwives employed at Ahmed Dhagah Mother and Child Health Clinic (MCH) and Hargeisa Group Hospital. The healthcare professionals asked the women to participate in the study when leaving the health facility after birth. In addition, local TBAs working in the area identified and approached women who they felt met the study criteria. These women were invited to take part in the study and were provided with verbal and written information about it. Participation was voluntary and the women were informed they could withdraw from the study without explanation. After giving their informed consent, participants signed a consent form with a thumbprint signature. An appointment for an interview to be held in the woman’s home was then arranged.

The interviews were conducted by the first three authors using a semi-structured interview guide. The questions focused on the aim of the study, which was to collect data on women’s choice of place of birth and to explore their experiences of these places. The questions were developed in English and then translated into Somali. They were then pilot tested on two of the participants which resulted in some minor clarifications to the guide before being used for the rest of the study. The questions included: Please tell me about your pregnancy and birth experiences. Please tell me why you decided on a home birth. How was your experience of antenatal care? Why did you decide on a facility birth? How was your experience of your home birth/facility-based birth? Who delivered your baby? The questions enabled the interviewees to speak freely about their experiences and the prompt to ‘please tell me more’ was used to encourage the informant to continue to tell their stories. The interviews were held in Somali, took about 45–60 minutes, were audio recorded and transcribed verbatim into Somali and then translated into English by the first two authors and double-checked by local University of Hargeisa staff.

### Analysis

The transcribed interviews (n = 25) were analysed inductively using qualitative content analysis [24]. In the *organization phase*, the transcripts were read and re-read to get a sense of the data as a whole. At this stage, two content areas were identified: Care experiences in facility-based birth and TBA supported home birth. Through a series of collaborative discussions within the team, the authors analyzed the original 123 pages of data. Text parts, sentences, or small paragraphs with the same meaning were labelled with a code and grouped together, based on similarities and differences in the texts, initially, at the hierarchical level closest to the original content, into sub-categories. These sub-categories were then grouped into more generic categories at a higher level of abstraction. What emerged from this process, the overarching main category, has been described in one sentence as: A lack of reproductive agency in facility-based births makes home births a first choice regardless of potential risks and medical needs. Examples of this analytical process are provided in Table 2. The outcome of the analysis was based on the contrast between women’s experience of facility-based births facilitated by healthcare professionals and home births facilitated by TBAs [24].

**Table 2.** Example of the analytical process

Content areas	Text parts, sentences, or small paragraphs	Codes	Sub-categories	Generic categories	Main category
Facility-based birth and care	*‘The young healthcare professional spoke to me in bad words.’* (Case 4)	Disrespectful care	Experiencing verbal abuse	Being met with disrespect	A lack of reproductive agency in facility-based births makes home births a first choice regardless of potential risks and medical needs
TBA supported home- delivery	*‘Xalimo [her TBA] was very caring for me and my children. She checked on me regularly and encouraged me to listen to my body and to let her know how I feel whenever I needed something.’ (Case 1)*	TBAs referring complications to health facilities	Being a constant presence	Preferring a trusting environment	

## Results

### The main category

The main category that emerged from the findings was that a lack of reproductive agency in facility-based births makes home births a first choice regardless of potential risks and medical needs. Women wish to exercise intentionality in their roles as wife and mother by choosing the environment when giving birth which best suits their needs and preferences as illustrated below. The organization of the results is presented in Table 3. A detailed description of the results is given under the headings and subheadings of the content areas and the generic categories.

**Table 3.** Content areas, categories and sub-categories, and overarching main category of the results

Content areas	Generic categories & sub-categories	Main category
Facility-based birth and care	**Searching for safety** - Needing specific medical competence - Using health education and examination results for guidance **Being met with disrespect** - Experiencing verbal abuse - Having it told, not explained	A lack of reproductive agency in facility-based births makes home births a first choice regardless of potential risks and medical needs
TBA supported home birth	**Preferring a trusting environment** - Being a constant presence - Sharing values and cultural understanding - Relying on TBAs to make referrals if needed **Feeling empowered and valued** - Being encouraged to take responsibility - Appreciating the TBA’s ‘background’ role	

### Facility-based birth and care

#### Searching for safety

##### Needing specific medical competence

The women who had had a facility-based birth had chosen this process because they knew that healthcare professionals were competent and skilled at obstetric emergencies. The women in this study were aware of the local maternity services available to them and appreciated the difference in function, competency, and capability between a healthcare professional at a health facility and a TBA. A facility-based birth, however, was only ever a second choice, selected when a TBA was not easily accessible, busy, or away when the woman went into labor:

I delivered in the health facility. I started labor pains one day when I was alone, so I tried to seek a TBA as the first choice for a home birth, but she was not present at that time, so as a second choice I saw a taxi driver that I knew, and I went to the health facility to give birth and, soon after I arrived at the health facility, I gave birth. All my family came to the health facility later. (Case 5)

Several women described how their experiences of care at a health facility had led them to decide to have their subsequent birth at home rather than at a facility. Choosing a facility-based birth was based on the desire to have an experienced midwife present while giving birth. Experiences of care at a health facility, however, prompted women decision to intentionally choose a home birth as their subsequent place of birth.

##### Using health education and examination results for guidance

Most of the women in this study had some form of antenatal care at a health facility, as part of the general measures in place in Somaliland designed to protect women’s health during pregnancy. The women described how most of the healthcare professionals working at the health facilities provided them with general health messages, explained some dietary requirements, and encouraged them to take medicine that would protect their health. The antenatal healthcare professionals provided them with assessments and a screening service. Based on the results of these tests, although it was not verbalized at the appointment with the health care professional, the women considered their specific circumstances and planned their place of birth:

I attended antenatal care, and they told me that I am normal and that my blood is fine … So, I thought if the baby is in a normal position, I could have the baby at home. That was why I decided to give birth at home. (Case 18)

However, even women who had had previous high-risk pregnancies that suggested they should be returnees to facility-based care, were still intentionally planning for a home birth, despite the evidence from their assessments and advice from healthcare professionals. Their strategy was that if they experienced abnormalities or emergencies at a home birth, they would then decide to utilize the maternity services at the health facility. One participant said: *‘The health facility is better when you face complications. There are trained healthcare professionals who manage your condition. There is care available that you cannot get at home.’* (Case 5)

#### Being met with disrespect

##### Experiencing verbal abuse

One woman who had experienced a complicated birth in a health facility said she felt medically safe although she was verbally abused:

I was in a very serious condition when I gave birth in the health facility, and the young healthcare professional spoke to me in bad words. The elderly woman that welcomed me and hoped to deliver me already her shift finished. That elderly woman made prayer and asked Allah to facilitate my birth. I was very happy to get support from that woman, but the younger healthcare professional, she assisted my birth, and I hated that. (Case 4)

Even though the women knew they might encounter a rushed environment, a lack of privacy, and possibly disrespectful and abusive behavior in a birthing process potentially fraught with medical interventions, they knew that a health facility-based birth was the safest option for them. One woman explained her experience of a facility-based birth: *‘I did not have any health problems, but still, I went to the health facility to give birth, but the healthcare professional slapped me.’* (Case 3)

##### Having it told, not explained

The women expressed disappointment with the quality of the health education, information, and advice they received from the healthcare professionals at the health facility. For example, pre-eclampsia was a prominent condition which the women in this study knew was one of the leading causes of maternal deaths. They found it careless of the healthcare professionals to simply diagnose high blood pressure and then fail to advise and explain the effects pre-eclampsia could have on labor if left untreated:

The healthcare professionals did not give me any health education or advice on how to manage my high blood pressure, but they measured my blood pressure and prescribed medication, and I bought my medication and used it regularly. (Case 3)

### TBA supported home-delivery

#### Preferring a trusting environment

##### Being a constant presence

According to the interviewees, TBAs supported women emotionally throughout their labor. The women valued this support a great deal and it motivated them to choose a home birth. One of the study participants recalled:

My TBA was always my supporter and although I delivered at home, she has always been available to me every minute of the birth she was with me. She never left my side and always asked me what I needed. She read something and stayed by my bedside all night. I felt her efforts to care for me the best she could. (Case 10)

TBAs providing accessible, individualized and respectful care, often lived nearby, either on the same street or in the same village.

##### Sharing values and cultural understanding

The participants felt that their TBAs shared the same values as they did and thus recognized the importance of dignity and understanding during the birthing process. The TBAs understood that they were a guest in the woman’s own home and that, above all else, their care needed to reflect the woman’s needs. One participant said: *‘Home and hospital birth are completely different. Women search for normality, someone that assists you, who is kind and keeps your dignity intact.’* (Case 4) TBAs also expected to be paid for their services although their fee was lower than that of a health facility, and they often took the woman’s financial situation into account. One woman pointed out that: *‘The TBA took a lower price for the birth than at the health facility. The hospitals have a fixed price.’* (Case 6) During the interviews, the women talked at length about the importance they placed on respect and privacy during labor.

##### Relying on TBAs to make referrals if needed

TBAs were, according to the participants, eager to avoid the complications associated with a ‘high risk pregnancy’. The women in this study who had had complicated pregnancies reported their TBAs constantly advising them to seek facility-based care so they would not be blamed if complications arose. One participant recalled:

The TBA encouraged me to go to the health facility and consult with the healthcare professionals. Whenever she visits us, she kept asking me if I went to the health facility this morning, if I check my blood pressure level. So, when I say yes, she always says all right, do not stop going to the health facility. Also, later I developed high blood pressure that is why I was very pleased that I was going to the health facility regularly. (Case 3)

#### Feeling empowered and valued

##### Being encouraged to take responsibility

The participants stated that during their home birth the TBAs encouraged them to listen and trust their bodies. This made the women feel empowered and in control of the events during their labor. They also appreciated the privacy of being in labor at home where they were able to take care of their other children. This expression of responsibility greatly reduced their anxiety and stress levels. One woman explained:

Xalimo [her TBA] was very caring for me and my children. She checked on me regularly and encouraged me to listen to my body and to let her know how I feel whenever I needed something. (Case 1)

##### Appreciating the TBA’s ‘background’ role

TBAs were able to practice ‘expectant management’. They were present, but in the background, watching the labor process, but allowing it to proceed on its own terms. The woman felt their TBA made them feel relaxed and empowered and did not rush or force the birthing process. The women thought that the healthcare professionals and TBAs had complementary roles and expertise. They felt greater collaboration between healthcare providers and TBAs could possibly improve maternity services in Somaliland. According to the women in this study, facility-based births disrupt family life because it forces them to be away from their children and the supportive and familiar environment of their home. Home births make all these concerns easy to manage:

It was my home and not a hospital. I slept in my warm bed at home but in the health facility, it is possible you feel cold and moist after childbirth. I slept with my baby, I got relaxed, and I had all the other children with me in my home. (Case 4)

Home births were described as a first choice because of the knowledge the women had from their previous experiences of care, and their ability to compare and rank those experiences according to their own values and intentions.

## Discussion

The results show that women perceived a lack of reproductive agency in facility-based births makes home births a first choice regardless of potential risks and medical needs. The fact that women felt empowered and in control in a home birth setting needs to be acknowledged, and these factors are important moving forward to improve the quality of maternal and newborn care in health facilities in accordance with WHO standards [26] and the QMNC framework (16).

A heavy workload for trained healthcare professionals with only minimal pay and the obligation to introduce complex and unfamiliar professional standards has the potential to create a culture and environment which are not always able to support a positive attitude towards women and their babies [27,28]. In this study, women simultaneously acknowledged the important work healthcare professionals did when treating maternal health emergencies at the health facility, yet consistently stated their preference for the caring values of a TBA for a normal labor and birth. As suggested by the QMNC framework, engaging with community, in this case through TBAs, in a midwife-led continuity of care model is an important element of an ideal care philosophy [16]. Altering maternal and child health clinics into midwife-led continuity of care models has been suggested as a component of birth preparedness and complication readiness for women in hard-to-reach areas [29]. For this philosophy to be realized, the number of midwives educated to global standards with the ability to provide holistic care within their local communities needs to be increased [30]. Our findings from Somaliland are supported by a growing literature that describes TBAs as a vital link between women and the healthcare professionals in their local maternity services [31]. A recent WHO report has shown that using shared values about birth, such as those expressed by women and TBAs, has been identified as a way to improve maternal and newborn health [32]. The WHO has also emphasized the importance of creating new roles and responsibilities for TBAs that link them to the formal healthcare system [33]. This is supported by a study that suggests TBAs could use their competence as a doula to provide cultural and psychosocial support during pregnancy and childbirth in Somaliland [34]. Another intervention, one supporting the results of this study, would be to provide healthcare professionals with training in respectful maternity care. This has been suggested in a review reporting that the maternity care healthcare professionals provide should be communicated to women and their families in a way that allows for planning, gives women the opportunity to choose, encourages them to ask questions, and is willing to disclose treatment results appropriately [35].

This study showed that healthcare professionals could have provided better health education and information to women at their antenatal check-ups, better informed them of the risks involved in pregnancy and birth, and offered them a better discussion of their birthing options. Such advice is especially important in fragile contexts like Somaliland. Women, like those in this study, are frequently obliged to carry out their own risk assessments and determine their own intentions during pregnancy. They are expected to interpret their own antenatal care assessments and, as a result, to determine for themselves their place of birth. Advice from trained health professionals communicated to them in a sensitive and collaborative way is crucial to achieve quality of care and to meet the need for a caring and convenient birth experience [26].

Midwives educated and trained according to ICM global standards, working in functional health systems and enabling environments can help reduce and prevent a majority of maternal and new-born deaths, and provide over 80% of essential sexual, reproductive, maternal, and new-born health services [17]. However, there is a lack of midwives in many low-resource settings, and important investments are needed to educate midwives, build their capacity to do research within their field, and take on leadership roles. They are the group with whom TBAs should work most closely in order to close the gap between home and facility-based births and assure responsive, relational, cost-effective, and patient-centred care [16]. Midwives are the key to establish and assure that midwife-led continuity of care models are implemented and scaled up [18]. It would be beneficial to further understand the roles of midwives in the Somaliland health systems to identify the core contextual factors within political and health systems that act as barriers or facilitators [36] in order to scale up access to high quality and acceptable maternal and new-born health care for women. Midwives competencies and capacity should be utilized at all levels in the health system using a multipronged approach improving leadership and management to develop and sustain midwife-led continuity of care models [37]. According to Yu et al. [30], the well-functioning midwife-led birthing centers studied in Australia result in significantly lower intervention rates, greater feelings of reproductive agency, and lower health system costs than carrying out normal births in hospitals, while avoiding some of the risks inherent in home births. Australia and Somaliland are obviously very different [38], but a similar study examining the specific circumstances of introducing midwife-led continuity of care in public health facilities in Somaliland would be informative, especially if carried out in combination with an investigation of the different functions and roles midwives and TBAs occupy, and how they could use their respective abilities to provide quality care for women and newborns. Addressing these challenges could encourage women to more willingly consider facility-based births, which would reduce adverse maternal and newborn health outcomes locally and globally.

### Strengths and limitations

This study captures the preferences of women in Somaliland when choosing their place of birth and why they chose to give birth at a health facility assisted by a healthcare professional or at home assisted by a TBA. Our preconceptions as midwives may have shaped our understanding in the analysis. By using the content analysis method with all authors involved in the analysis process, we aimed to provide confidence to the reader of the reliability of the findings [24,38]. The key strength of this study was therefore the research process and the personal interviews which gave voice to some of Somaliland’s women. It is the first study of its kind in the Somali region and provides vital insights into women’s choice of place of birth.

Despite the high credibility of its findings [24,37], this study has a series of limitations that affect its transferability. Transferability refers to whether the findings have applicability in other contexts [24,39]. The findings in this study have been confirmed in other studies in the field. This strengthens dependability [24,39], showing that the findings are consistent. The main limitation of this study is that multipara women might, because of their age and experience, be more intentional than younger and less experienced primipara women when choosing their place of birth. This is evident in a previous health survey [40] that shows how agency with regards to women’s sexual and reproductive health decisions seem to increase with age and childbirth. Women’s choices of place of birth are influenced by many personal, social, and healthcare service factors. The findings of this study might thus be shaped by the participants’ age, parity, knowledge, and experience. In addition, the financial status of the women might have influenced their choices [41]. Because women in our study were from a relatively poor socio-demographic background, they might have been more inclined to choose a home birth because of financial considerations. The findings outlined here, therefore, should be transferred with caution to primipara women or women from secure economic backgrounds. Further research is needed to define and test a context specific midwife-led continuity of care model to be scaled up at public health facilities. The function and role of TBAs and the possibility of linking TBAs more closely to the healthcare system, and possible barriers to such a development, are further suggested. Above all else, this research should be carried out within the framework of a long-term sustainable plan to assure quality and equal access to maternal healthcare in Somaliland based on the WHO standards for improving maternal and newborn quality of care in health facilities [26]. Even if there is existing literature examining women’s choice of place for delivery [7,10,11,14], this is one of the few studies coming from a fragile post-conflict setting.

## Conclusion and implications

To meet women’s needs and preferences in Somaliland, further investments are needed to strengthen the midwifery profession and to define and test a context specific midwife-led continuity of care model to be scaled up at public health facilities. A dialogue to create new roles and responsibilities for the TBAs who attend most home births is further needed to link them to the formal healthcare system and assure timely healthcare seeking during pregnancy and birth.

The implications of this study are to:

- Increase the number of midwives, strengthen their role in midwife-led continuity of care models, and better organize and integrate them into the maternity care system at all levels
- Create new roles and responsibilities for TBAs that link them to the formal healthcare system, and assure connections to the community
- Provide healthcare professionals with a training package on respectful maternity care that would help to end the mistreatment of women in maternity services
- Use context specific, scientific evidence as a foundation to develop a policy on quality maternity care for the Somaliland maternity services to be used in political decision making and investments.

## Paper context

Women in low resource settings choose home births with untrained, traditional birth attendants in preference to health authorities’ facility-based maternity services. This study provides a valuable description of why multipara women in low-resource settings choose a home birth.

To meet women’s needs and preferences in Somaliland, further investments are needed to strengthen the midwifery profession and to define and test a context specific midwife-led continuity of care model to be scaled up at public health facilities. A dialogue to create new roles and responsibilities for the TBAs who attend most home births is further needed to link them to the formal healthcare system and assure timely healthcare seeking during pregnancy and birth.
